# Cerebral microdialysis for detection of bacterial meningitis in aneurysmal subarachnoid hemorrhage patients: a cohort study

**DOI:** 10.1186/cc7689

**Published:** 2009-01-20

**Authors:** Florian Schlenk, Katja Frieler, Alexandra Nagel, Peter Vajkoczy, Asita S Sarrafzadeh

**Affiliations:** 1 Department of Neurosurgery, Charité – Universitätsmedizin Berlin, Campus Virchow Klinikum, Augustenburger Platz 1, 13353 Berlin, Germany; 2 Institute of Biometry and Clinical Epidemiology, Charité – Universitätsmedizin Berlin, Charitéplatz 1, 10098 Berlin, Germany

## Abstract

**Introduction:**

Bacterial meningitis (BM) is a severe complication in patients with aneurysmal subarachnoid haemorrhage (SAH). Clinical signs of meningitis are often masked by SAH-related symptoms, and routine cerebrospinal fluid (CSF) analysis fails to indicate BM. Microdialysis (MD) is a technique for monitoring cerebral metabolism in patients with SAH. A cohort study was performed to investigate the value of MD for the diagnosis of BM.

**Methods:**

Retrospectively, 167 patients with SAH in an ongoing investigation on cerebral metabolism monitored by MD were analysed for the presence of BM and related MD changes. Diagnosis of BM was based on microbiological CSF culture or clinical symptoms responding to antibiotic treatment, combined with an increased CSF cell count and/or fever. Levels of MD parameters before and after diagnosis of BM were analysed and compared with the spontaneous course in controls.

**Results:**

BM developed in 20 patients, of which 12 underwent MD monitoring at the time of diagnosis. A control group was formed using 147 patients with SAH not developing meningitis. On the day BM was diagnosed, cerebral glucose was lower compared with the value three days before (p = 0.012), and the extent of decrease was significantly higher than in controls (p = 0.044). A decrease in cerebral glucose by 1 mmol/L combined with the presence of fever ≥ 38°C indicated BM with a sensitivity of 69% and a specificity of 80%. CSF chemistry failed to indicate BM, but the cell count increased during the days before diagnosis (p < 0.05).

**Conclusions:**

A decrease in MD glucose combined with the presence of fever detected BM with acceptable sensitivity and specificity, while CSF chemistry failed to indicate BM. In patients with SAH where CSF cell count is not available or helpful, MD might serve as an adjunct criterion for early diagnosis of BM.

## Introduction

Bacterial meningitis (BM) is a severe and cost-intensive complication in patients with aneurysmal subarachnoid haemorrhage (SAH) and requires immediate treatment. Although appropriate therapy with potent antibiotics is available, BM continues to be associated with a prolonged stay in the intensive care unit (ICU) and high morbidity [1]. External drainage of cerebrospinal fluid (CSF), frequently applied especially in patients with high-grade SAH, may raise the risk of infections of the central nervous system (CNS). The diagnosis is difficult in patients with SAH because the clinical signs of meningits are often masked by SAH-related symptoms. Furthermore, an alteration in CSF composition known as aseptic meningitis is frequent after SAH and cannot reliably be distinguished from CSF changes caused by BM [2]. Routine microbiological and chemical analysis of CSF for the prediction or diagnosis of BM failed in patients with external drains [3]. Considering these restrictions and the risks associated with a delayed diagnosis of BM, additional tools to facilitate the early diagnosis of BM after SAH would be desirable.

Cerebral microdialysis (MD), an advanced neuromonitoring technique, gives online information on the metabolic state of the injured brain. This technique is mainly used to detect ischaemia-related changes for early diagnosis of symptomatic vasospasm in patients with SAH [4-6]. Additionally, it allows quantification of interleukins and thereby provides analysis of immunological processes within the brain, which may allow earlier therapeutic measures in the beginning of immunoreactive cascades and thereby improve outcome in these patients [7]. For investigating intracranial infections, the MD technique has only been used in animal models and two case studies in humans [8,9]. During experimental pneumococcal meningitis, a decrease in cortical glucose levels and an increase in local lactate production in the brain were observed [10]. Hence, the present study aimed to evaluate whether cerebral MD allows – besides the monitoring of ischaemia-related changes – the early detection of BM, and to assess its significance compared with CSF analysis and clinical changes.

## Materials and methods

### Patient population

Both this study and the underlying prospective investigation on cerebral metabolism were approved by the Local Research Ethics Committee at Charité Campus Virchow Medical Center, in accordance with the Declaration of Helsinki as revised in Edinburgh in October 2000. Written informed consent was obtained from the patient or their nearest family relative.

### Patients characteristics and management

Retrospectively, 170 consecutive patients with SAH, who were part of an ongoing study on cerebral metabolism monitored by bedside MD, were selected for analysis of MD changes related to meningitis. All the patients had confirmed aneurysmal SAH and underwent surgical treatment. Distribution and pattern of the haemorrhage were graded as proposed by Fisher and colleagues [11], and clinical presentation was graded according to the World Federation of Neurological Surgeons (WFNS) scale [12]. Neurological outcome after six months was assessed using the Glasgow outcome scale [13]. Demographic and clinical data are summarised in Table 1. In the computed tomography (CT) scans of the patients, the exact position of the MD catheter was reviewed. Three of the 170 patients were excluded because the catheter was situated close to an intracerebral haemorrhage, because in this area lactate and glutamate levels are known to be elevated [14]. The remaining 167 patients were included in this analysis.

**Table 1:** Demographic and clinical characteristics of 167 patients following aneurysmal subarachnoid haemorrhage

	**All patients** (n = 167)	**Meningitis** (n = 20)	**Controls** (n = 147)	p
Age	50.8 ± 12.6	53.5 ± 9.7	50.4 ± 13.0	p = 0.292
Gender: male/female	46/121	5/15	41/106	p = 0.802
Admission WFNS grade	2.7 ± 1.6	3.6 ± 1.3	2.6 ± 1.6	p = 0.009
0	3 (2%)	0 (0%)	3 (2%)	
I	58 (35%)	2 (10%)	56 (38%)	
II	21 (13%)	3 (15%)	18 (12%)	
III	18 (11%)	2 (10%)	16 (11%)	
IV	38 (23%)	8 (40%)	30 (20%)	
V	29 (17%)	5 (25%)	24 (16%)	
Clinical group				p = 0.867
Asymptomatic	66 (40%)	8 (40%)	58 (40%)	
AFND	58 (35%)	6 (30%)	52 (35%)	
DIND	43 (26%)	6 (30%)	37 (25%)	
Time SAH – surgery (hours)	45.0 ± 74.4	61.0 ± 133.3	42.8 ± 62.3	p = 0.519
Fisher-score	3.0 ± 1.0	3.5 ± 0.6	3.0 ± 1.0	p = 0.059
Duration of microdialysis (hrs)	165.4 ± 83.8	185.2 ± 96.8	162.8 ± 81.9	p = 0.252
Presence of CSF drainage	77 (46%)	18 (90%)	59 (40%)	p = 0.001
ICU stay (days)	15.1 ± 9.3	20.6 ± 6.9	14.3 ± 9.4	p = 0.003
GOS at 6 months after SAH	3.8 ± 1.4	4.0 ± 1.2	3.8 ± 1.4	p = 0.889
Mortality rate at 6 months	20 (13%)	1 (6%)	19 (14%)	p = 0.322

P values are given for comparison of patients developing meningitis and controls (calculated by Mann-Whitney-U-Test or, for dichotomous data, by chi-squared test, exact versions).

AFND = acute focal neurological deficit; CSF = cerebrospinal fluid; DIND = delayed ischaemic neurological deficit; GOS = Glasgow outcome scale; ICU = intensive care unit; SAH = subarachnoid haemorrhage; WFNS = World Federation of Neurological Surgeons.

### Bedside microdialysis

In all patients, cerebral metabolism had been measured by an MD catheter (CMA 70, CMA, Solna, Sweden); membrane length 10 mm; molecular weight limit of 20 or 100 kD) in the brain parenchyma of the corresponding vascular territory of the aneurysm, with the catheter tip being located about 1.5 cm from dura level. The correct positioning of the catheter tip within the vascular territory of the occluded aneurysm was verified postoperatively by CT. Catheters were perfused with sterile Ringer's solution at a flow rate of 0.3 μl/minute. The estimated recovery for the system is 0.65 to 0.72 (recovery in the two different catheter types is comparable for molecules up to 20 kD) [15,16]. On the outlet tube, perfusates were collected in microvials and analysed hourly at the bedside for parameters of energy metabolism (glucose; pyruvate; lactate; lactate/pyruvate (L/P) ratio; lactate/glucose (L/G) ratio) as well as glycerol and glutamate, in a mobile photometric, enzyme-kinetic analyser (CMA 600, CMA, Solna, Sweden). MD was performed for 7 to 10 days after SAH, and daily medians of the microdialysate concentrations were calculated for each patient.

### Diagnosis of bacterial meningitis and evaluation of related microdialysate changes

The ICU records of the patients measured by MD were examined for the presence of fever of 38°C or above, the results of microbiological and chemical CSF analysis, and the daily records of the clinical state. Routine chemical CSF analysis in patients with external drains included daily evaluation of cell count, protein, glucose and lactate concentrations, as well as microbiological culture in case of fever, changes in CSF chemistry and unclear neurological deterioration. In patients without CSF drainage and suspicion of meningitis, CSF samples were obtained by lumbar puncture. Patients with diagnosed BM were recorded. In this retrospective study, patients were accepted as having BM when the diagnosis was based on either the presence of bacteria in CSF culture or on clinical symptoms that rapidly responded to antibiotics typical for BM treatment, in combination with increased CSF cell count and/or fever of 38°C or above.

For assessment of BM-related changes in the composition of cerebral extracellular fluid, the daily medians of microdialysate concentrations were evaluated for three days preceeding the diagnosis of BM up to day two after diagnosis. The same analysis was performed for CSF cell count and concentrations of glucose, lactate and protein, as well as blood glucose concentrations. MD changes during three days before diagnosis of BM were compared with the spontaneous course in the control group. In this analysis, only meningitis patients with a complete MD dataset for this three-day period were included. Because the onset of meningitis differed substantially between patients, randomly selected periods of three days were chosen in controls instead of the mean interval between haemorrhage and meningitis. These periods were selected by computer individually for each patient. Several repetitions of this procedure with different, randomly selected intervals brought comparable results.

### Data analysis

The MD and CSF data were collected during days 1 to 10 after SAH. Data in tables and text are expressed as mean ± standard deviation, if not otherwise specified. Statistical analysis was based on each patient's daily median values for each MD variable and on daily CSF samples. Average microdialysate values given for a group of patients were established calculating the mean value of the patients' individual daily medians for the MD parameters. Between group comparisons were performed by nonparametric Mann-Whitney U tests and chi-squared tests for dichotomous data (exact versions). Analysis of sequential data over time was performed using Wilcoxon signed-rank test. Sensitivity and specificity of changes in MD parameters for diagnosis of BM were evaluated in all the patients undergoing MD monitoring at the time of meningitis. They were calculated for MD changes alone and in combination with fever of 38°C of above. Results were displayed by receiver operating curves, and the cutoff value for MD changes that showed the highest sum of sensitivity and specificity was calculated. Statistics were calculated using SPSS 14.0 (SPSS Inc., Chicago, IL, USA) and R, Version 2.6.0 (The R Foundation for Statistical Computing, Vienna, Austria). Differences were considered statistically significant at p < 0.05.

## Results

### Patients

Results from 1.025 daily MD medians and 77 CSF samples in 167 patients were analysed. Twenty patients (12%) had been diagnosed with BM by the physician in charge. All of them fit this study's criteria for meningitis and were included. At the time of meningitis 12 of the patients were undergoing MD monitoring and in eight patients MD values were available for the complete three-day-period before diagnosis of BM.

In 10 patients (50%), diagnosis of BM was based on microbiological CSF findings (six patients had MD monitoring at the time of diagnosis). Microbiological characteristics of these patients are shown in Table 2. In a further 10 patients, diagnosis was based on CSF changes and clinical symptoms responding to antibiotic treatment typically used for BM (four patients had MD monitoring at the time of diagnosis). One patient had already received antibiotic treatment before diagnosis of BM (in this case, BM was confirmed by microbiology). CSF values were available for 14 patients with BM. A control group was formed using 147 patients with SAH who did not develop any CNS infection during the observation period.

**Table 2:** Microbiological characteristics of 10 patients with bacterial meningitis after aneurysmal subarachnoid haemorrhage.

**Causative agent**	**Number of patients**
*Staphylococcus aureus*	4
Coagulase-negative *Staphylococci*	6
*S. epidermidis*	2
*S. warneri*	1
*S. capitis*	1
*Acinetobacter baumannii*	1
*Escherichia coli*	1

Bacteria were identified by microbiological culture of cerebrospinal fluid. In some patients, more than one organism was found.

### Risk factors and outcome in patients with SAH developing bacterial meningitis

Patients developing BM were more severely affected by the haemorrhage than controls (WFNS grade: p = 0.01). This is most likely to be due to the fact that in patients with high-grade SAH a CSF drainage, known as a risk factor for BM, was present more frequently than in controls. Almost all patients with BM had CSF drainage (90%), but only 40% of patients not developing CNS infections had a CSF drainage (p < 0.001). On the day of diagnosis of BM, a CSF drainage had been present for 7.63 ± 3.2 days (range 2 to 14 days), and the actual drainage had been present for 6.69 ± 3.4 days (range 1 to 14 days).

**Figure 1:**
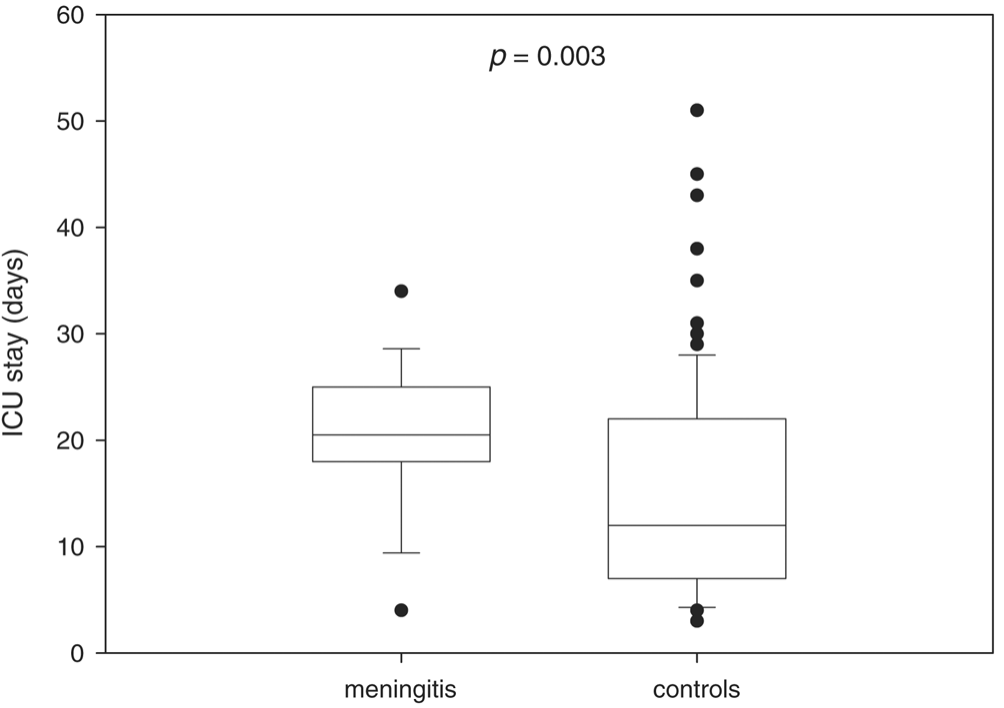
**Length of stay in the intensive care unit (ICU) in patients with aneurysmal subarachnoid haemorrhage (SAH)**. Box plot presenting ICU stay in patients with SAH and meningitis, and in patients with SAH and no infections of the central nervous system. Patients who died during the ICU stay were excluded. Boxes represent 25^th^, 50^th ^and 75^th ^percentiles; dots mark the outlier values. P values were calculated using Mann-Whitney U test (exact version).

In this small group of patients with BM, mortality rate or neurological outcome at six months after SAH did not differ significantly from controls. ICU stay was prolonged by 38% in patients with BM (20.4 ± 7.6 vs. 14.8 ± 9.7 days after excluding patients who died during ICU stay; p = 0.003; Figure 1).

### CSF and MD changes related to bacterial meningitis

During the time span from three days before to two days after diagnosis of BM, no significant changes in CSF glucose, lactate or protein concentrations were observed. CSF cell count, however, was significantly higher on the day of diagnosis than three (p = 0.028) and two (p = 0.01) days before. It should be mentioned that in some patients the diagnosis of BM was based on this elevation in CSF cell count, so the role of this parameter as an independent indicator of BM cannot be reliably evaluated in this study.

**Figure 2:**
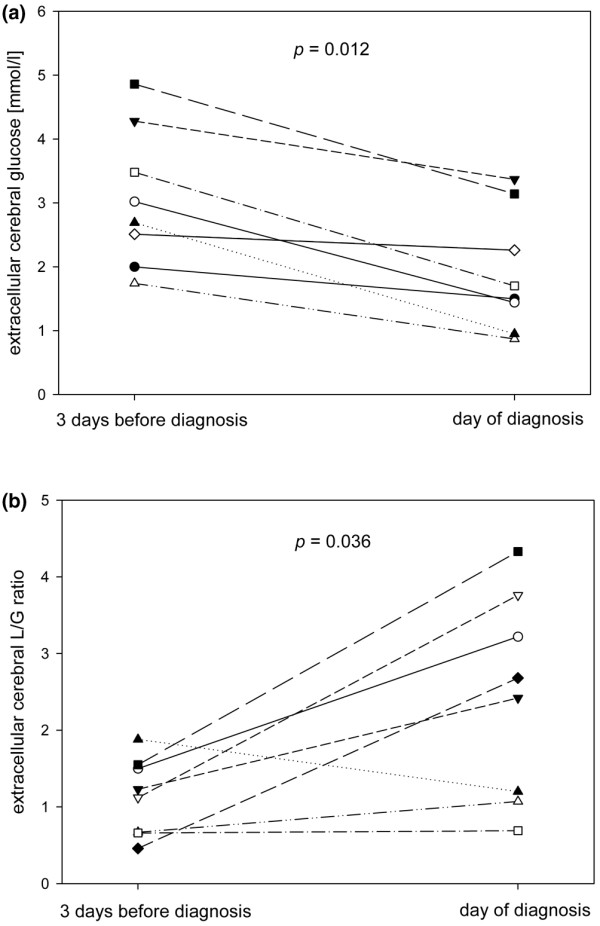
**Cerebral extracellular glucose and lactate/glucose ratio in patients with aneurysmal subarachnoid haemorrhage (SAH) and bacterial meningitis**. Individual courses of **(a)** cerebral extracellular glucose and **(b)** lactate/glucose (L/G) ratio in patients with SAH and bacterial meningitis. Daily median microdialysate concentrations on three days before diagnosis of meningitis and on the day of diagnosis are presented for those patients with a complete dataset for that period. Each line represents one individual patient. P values were calculated using Wilcoxon signed-rank test.

**Figure 3:**
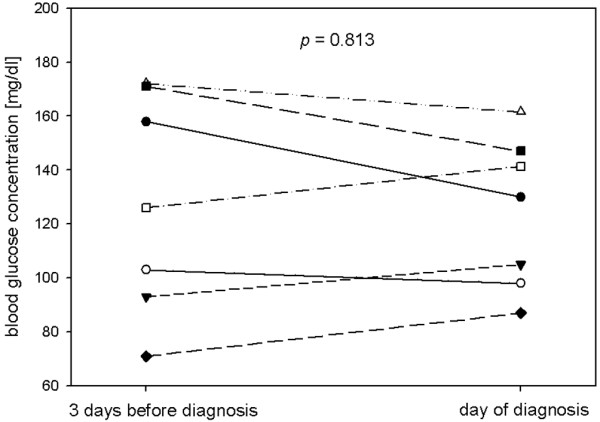
**Blood glucose in patients with aneurysmal subarachnoid haemorrhage (SAH) and bacterial meningitis**. Individual courses of blood glucose in patients with SAH and bacterial meningitis. Concentrations on three days before diagnosis of meningitis and on the day of diagnosis are presented for those patients with a complete dataset for that period. Each line represents one individual patient. P values were calculated using Wilcoxon signed-rank test.

In cerebral extracellular fluid analysed by MD, changes in glucose and L/G ratio were observed. On the day BM was diagnosed, cerebral glucose was lower (p = 0.012) and the L/G ratio higher (p = 0.036) compared with three days before (Figure 2). The extent of decrease in glucose was significantly higher than in controls (p = 0.044), while the course of L/G ratio did not differ significantly from the control group (MD changes at three days before diagnosis of BM, compared with randomly selected periods of three days in controls). The other measured parameters showed no significant changes during the three days before diagnosis of BM. During this period of time, there was no significant change in blood glucose concentrations in this group of patients, which would naturally have to be considered as a cause of changes in cerebral glucose, independent of meningitis (Figure 3).

**Figure 4:**
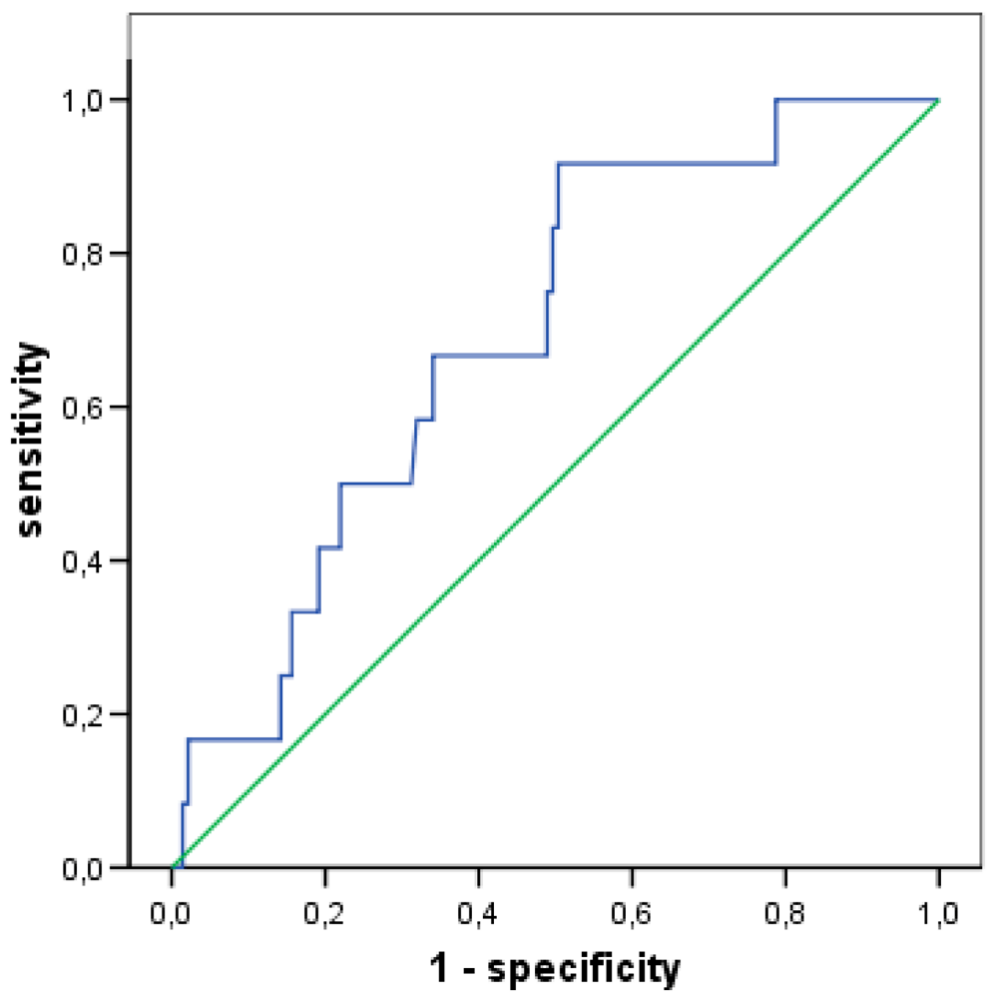
**Receiver operating curve (ROC) showing sensitivity and specificity of cerebral glucose changes for diagnosis of bacterial meningitis**. Twelve patients with aneurysmal subarachnoid haemorrhage (SAH) and meningitis undergoing microdialysis measurement at the time of diagnosis and 141 without infections of the central nervous system were included. Data are given for maximum glucose decrease over variable time spans within the observation period. Area under the curve (AUC) = 0.69, standard error = 0.07 (p = 0.026 compared with null hypothesis with AUC = 0.5). Blue line: Sensitivity and specificity of cerebral glucose changes for diagnosis of meningitis; green line: sensitivity and specificity for null hypothesis.

**Figure 5:**
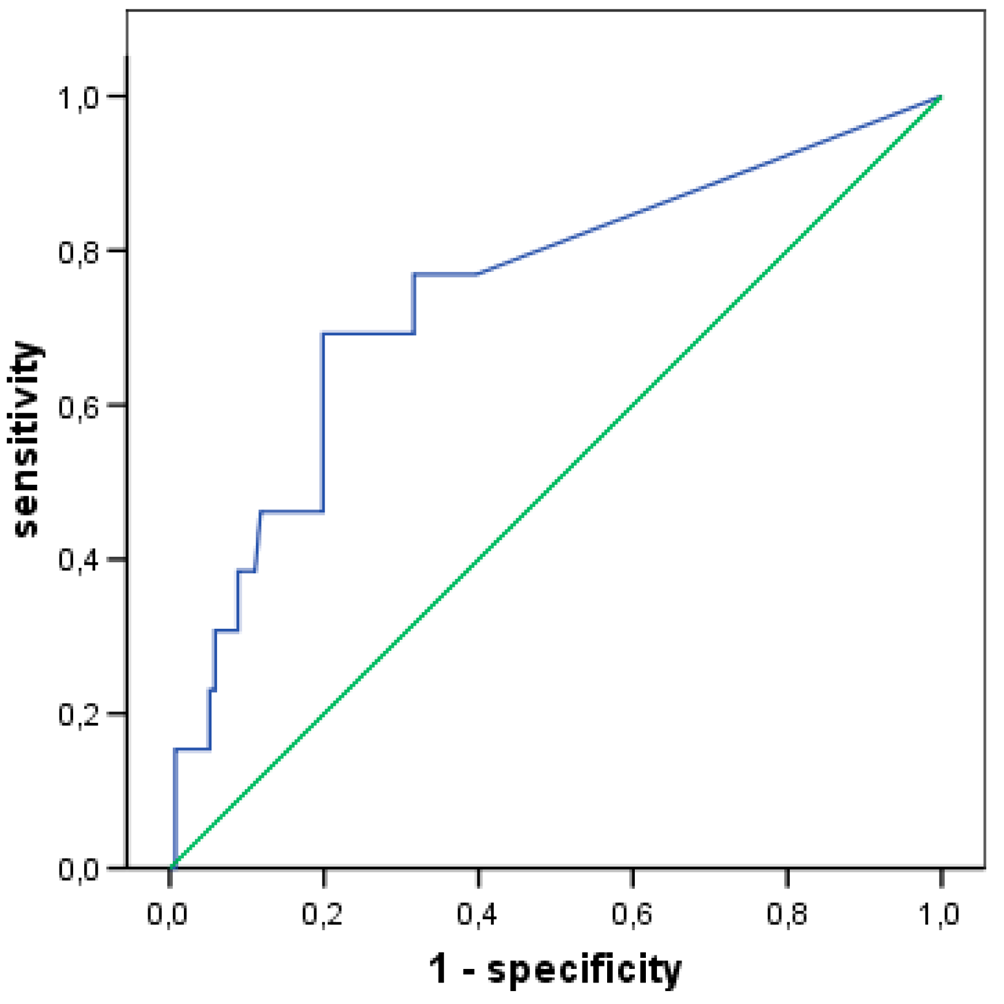
**Receiver operating curve (ROC) showing sensitivity and specificity of cerebral glucose changes combined with presence of fever ≥ 38°C for diagnosis of bacterial meningitis**. Twelve patients with aneurysmal subarachnoid haemorrhage (SAH) and meningitis undergoing microdialysis measurement at the time of diagnosis and 136 without infections of the central nervous system were included. Data are given for maximum glucose decrease over variable time spans within the observation period. Area under the curve (AUC) = 0.74, standard error = 0.08 (p = 0.004 compared with null hypothesis with AUC = 0.5). Blue line: Sensitivity and specificity of cerebral glucose changes with fever for diagnosis of meningitis; green line: sensitivity and specificity for null hypothesis.

The cutoff value for a decrease in MD glucose that showed the highest sum of sensitivity and specificity for indication of BM was 1 mmol/L. The diagnostic power of cerebral glucose changes and fever is summarised in Table 3. A decrease of 1 mmol/L over any time span identified BM with a sensitivity of 92% and a specificity of 50% (Figure 4), while the presence of fever (≥ 38°C) together with a glucose decrease of 1 mmol/L reached a sensitivity of 69% and a specificity of 80% (Figure 5).

**Table 3:** Diagnostic value of cerebral extracellular glucose and fever for bacterial meningitis in patients with aneurysmal subarachnoid haemorrhage

	**Glucose decrease ≥ 1 mmol/L**	**Body temperature ≥ 38°C**	**Glucose decrease ≥ 1 mmol/L AND body temperature ≥ 38°C**
**Sensitivity**	92%	83%	69%
**Specificity**	50%	60%	80%
			
**Positive predictive value**	20.1%	22.1%	32.0%
**Negative predictive value**	97.9%	96.6%	95.0%

The results are displayed for a decrease in cerebral extracellular glucose of ≥ 1 mmol/L over variable time spans within the observation period, and for presence of fever ≥ 38°C. Positive and negative predictive values were calculated for a prevalence of meningitis of 12%, as found in the present study.

## Discussion

This study aimed to assess the value of cerebral MD for diagnosis of BM in patients with SAH. There are three major findings. First, ICU stay was prolonged by an average of six days in patients with SAH and BM. Second, CSF chemistry failed to indicate BM in the observed patients, although CSF cell count showed a significant increase. Third, a decrease in cerebral glucose by 1 mmol/L measured by MD combined with the presence of fever detected BM with a sensitivity of 69% and specificity of 80%.

### Significance of CNS infections in patients with SAH

Hydrocephalus is a frequent complication after SAH and is usually treated by external CSF drainage, so this patient group is at risk of CNS infections [17]. The MD catheter itself might also be a port of entry for bacteria, but MD monitoring has not been reported to be associated with an increased risk of CNS infections [4,18,19]. With an incidence of between 1 and 9 per 100 patients, CNS infections are among the most commonly documented infections in neurointensive care units [17,20-22]. In a recent study in a large SAH population, meningitis/ventriculitis occurred in 5% of patients and was the fourth most common nosocomial infection after pneumonia (20%), urinary tract infections (13%) and bloodstream infections (8%) [1]. In that study, older age, greater severity of SAH, presence of intraventricular haemorrhage or ventricular drains, and longer ICU stay were identified as risk factors for CNS infection. An increased rate of death or severe disability could not be shown, consistent with another investigation among 638 patients treated with CSF drainage [17]. Infection rates appear not to be reduced by routine exchange of the drain, but only by shortening the total draining time [23,24].

### Diagnosis of bacterial meningitis in patients with SAH

Diagnosis of BM is usually based on clinical symptoms and changes in CSF composition [25]. These are, however, of limited predictive value even in patients without additional neurological disease [26]. In patients with SAH, clinical symptoms are even more unreliable, because most of the typical signs of meningitis such as headache, nuchal rigidity and altered mental status cannot safely be distinguished from SAH-related symptoms. Fever is also an unreliable diagnostic marker. Due to impaired cerebral temperature regulation after SAH, elevated body temperatures can also occur spontaneously or be absent in cases of severe infection. Additionally, respiratory and urinary tract infections are more frequent in these patients than BM, so suspect of meningitis is often raised late if there are no typical clinical or CSF findings. However, CSF composition is frequently altered by biochemical reactions to the subarachnoid blood and therefore unreliable. Typical CSF changes can also be absent if patients are already receiving antibiotic treatment for other infections such as pneumonia. In our study, only cell count showed a significant BM-related increase, but cannot reliably be evaluated because diagnosis of BM was based on that parameter in some patients. In general, CSF leucocyte count after SAH is often altered by blood cells and can even be impossible to evaluate in extremely bloody CSF. The insufficiency of CSF analysis for diagnosis of BM in these patients was strikingly demonstrated by Schade and colleagues [3]. In a study investigating the value of routine CSF analysis for diagnosis of BM in patients with external drains, it was not possible to establish a cutoff value with a sensitivity and specificity of at least 60% for any of the parameters of leucocyte count, protein, glucose and CSF/blood glucose ratio. Gram stain of CSF samples reached a specificity of 99.9%, but sensitivity was as low as 39.8% [3]. Still, routine CSF analysis remains recommended in patients with external drains, but these data clearly illustrate the need for additional tools to guide the diagnosis of bacterial meningitis in patients with SAH.

### Changes in MD parameters during bacterial meningitis

MD is used for monitoring cerebral metabolism in patients with SAH. Among others, glucose and lactate levels are usually assessed. The CSF concentrations of these markers are routinely used for diagnosis of BM, so their cerebral extracellular concentrations can also be expected to indicate BM-related changes. So far, MD data in meningitis are only available from experimental studies carried out mainly for measurement of drug penetration or pathophysiology of BM-related neuronal injury, and from two case reports in humans.

An experimental study in rabbits with pneumococcal meningitis revealed a cerebral increase in lactate and a decrease in glucose, and the authors conclude that BM leads to anaerobic glycolysis with increased lactate production within the brain [10]. A trial evaluating the permeability of antibiotics across the blood-brain barrier showed an increase in cerebral lactate and glutamate, and a slight increase in glycerol towards the end of the 24-hour observation period. Extracellular glucose was not measured in that study [27]. In an investigation of MD concentrations of several amino acids in a rabbit model of pneumococcal meningitis, glutamate was greatly elevated and was interpreted as an indicator that excitotoxic neuronal injury may play a role in BM [28]. In rabbits undergoing *Escherichia coli *meningitis, significant but late elevations in the excitatory amino acids aspartate and glutamate as well as in the inhibitory neurotransmitters γ-amino butyric acid and taurine were observed, which the authors assume – in contrast to the preceeding study – to be caused by cerebral ischaemia because of septic shock rather than the meningitis itself [29].

The available patient case reports focus on pathophysiological disturbances in the course of BM, not on early metabolic changes that could be used to guide diagnosis. In a patient with BM after a severe head injury, cerebral glucose decreased below the detection limit, combined with moderately high lactate levels and a marked increase in glutamate and pyruvate [8]. In another case of MD monitoring during meningoencephalitis, L/P ratio and glycerol were reported to remain stable, the other parameters were not mentioned [9].

Considering these results, a reduction in cerebral glucose and an increase in lactate are most likely to be expected during BM, eventually combined with an increase in glutamate. In the present study, glucose decreased markedly in the days before diagnosis of BM, but no significant changes in lactate, pyruvate or L/P ratio were noted. This might be due to the fact that the lactate levels and the L/P ratio take different courses according to the patient's clinical group (presence or absence of acute/delayed neurological deficits), so BM-related changes might have been obliterated by these alterations [4]. A significant change in glutamate was also not noted. A possible meningitis-related increase might have been masked by the continuous glutamate decrease, which is part of the spontaneous course after SAH in the absence of ischaemia, or have been oppressed by antibiotic treatment.

In how far the meningitis-related glucose depletion might limit the cerebral energy supply and require specific treatment remains speculative, although low cerebral glucose has been shown to be associated with unfavourable outcome in traumatic brain injury and severe metabolic derangements in patients with SAH [30,31].

In our study, a decrease in cerebral glucose of 1 mmol/L had a high sensitivity (92%) but low specificity (50%) for the diagnosis of BM. Together with the presence of fever of 38°C or above, acceptable sensitivity (69%) and specificity (80%) were reached. This reduction in sensitivity when temperature was included as a diagnostic factor can be explained by the absence of fever in some patients with possible impaired temperature regulation after SAH. As mentioned before, none of the standard CSF parameters used for the diagnosis of BM achieves a sensitivity or specificity of at least 60% in patients with external ventricular drains [3]. Considering this, cerebral MD might prove to be a useful tool not only to give pathophysiological insight in meningitis-related changes in brain metabolism, but also to facilitate diagnosis of BM in patients undergoing MD monitoring for the detection of symptomatic vasospasm, and thereby allowing earlier diagnosis and treatment to improve outcome. However, its diagnostic value will have to be confirmed prospectively in a larger patient population.

### Limitations of this study

There are several limitations of this study. Above all, the retrospective design did not allow exact definitions of the criteria for diagnosis of BM, and some patients might have been missed. No other infections simultaneously to BM were registered in the patient records. However, retrospectively, it cannot be excluded that another infection was present in some cases. Neither can the diagnostic value of the CSF cell count for BM be evaluated in this study, because diagnosis of meningitis had in some patients been based on this parameter which would bias the statistical analysis. Furthermore, MD is a regional method with the volume of brain tissue monitored by the catheter covering only a few millimeters from the membrane, and metabolic processes in the brain tissue affected by SAH might not always be representative for the whole brain. Finally, in spite of the large number of patients, only a few individuals sustained BM during the period of MD monitoring. Therefore, this work should be considered a pilot study, and the reliability of cerebral metabolic changes measured by MD for diagnosis or prediction of BM in patients with SAH will have to be confirmed prospectively in a substantially larger study population. Nevertheless, this is the first study to our knowledge evaluating cerebral metabolic changes during BM in humans.

## Conclusions

BM is a relevant complication in patients with SAH, and ICU stay was prolonged by an average of 5.6 days in patients with SAH and BM. The validity of clinical signs and routine CSF analysis for the diagnosis of BM in patients with SAH is limited. A decrease in MD glucose combined with the presence of fever indicated BM with acceptable sensitivity and specificity, while CSF chemistry including leucocyte count, protein and glucose failed to indicate BM. In patients with SAH where CSF cell count is not available or helpful, MD changes may serve as an adjunct criterion for early diagnosis of BM. The power of this study is limited because of the small patient number and the retrospective design, so the results will have to be confirmed in a larger, prospective study.

## Key messages

• BM occurred in 12% of patients with SAH

• In patients with SAH developing BM, ICU stay was prolonged by an average of 5.6 days

• CSF chemistry failed to indicate BM after SAH

• A decrease in MD glucose levels by 1 mmol/L, combined with fever of 38°C or above indicated BM with a sensitivity of 69% and a specificity of 80%

## Abbreviations

BM: bacterial meningitis; CNS: central nervous system; CSF: cerebrospinal fluid; CT: computer tomography; ICU: intensive care unit; L/G: lactate/glucose; L/P: lactate/pyruvate; MD: microdialysis; SAH: subarachnoid haemorrhage; WFNS: World Federation of Neurological Surgeons.

## Competing interests

The authors declare that they have no competing interests.

## Authors' contributions

FS participated in the design of the study and performed part of the statistical analysis, created the tables and figures and drafted the manuscript. He also managed the MD monitoring and collected the MD and blood samples from some of the patients and compiled the data from the patients' files. KF performed part of the statistical analysis. AN collected the MD and blood samples from some of the patients and compiled the data from the patients' files. PV supervised MD monitoring and revised the manuscript for important intellectual content. AS conceived of the study and, as the project leader, was responsible for the design, coordination and data interpretation and participated in drafting the manuscript.
